# Rotation of Hexamethylenetetramine Molecules Induces Reversible Electromagnetic Coupling Properties in Isothiocyanato-Nickel Complexes

**DOI:** 10.3390/ijms26094050

**Published:** 2025-04-25

**Authors:** Adila Abuduheni, Leilei Zhou, Yubing Yao, Yang Liu, Hongzhi Hu, Zunqi Liu

**Affiliations:** 1 Chemistry and Chemical Engineering College, Xinjiang Agricultural University, Urumqi 830052, China; 17799751675@163.com (A.A.); 18581355615@163.com (L.Z.); huhongzhi305@163.com (H.H.); 2 School of Computer and Information Engineering, Xinjiang Agricultural University, Urumqi 830052, China; yby2025xjnd@163.com; 3 Xinjiang Sub-Center National Engineering Research Center of Novel Equipment for Polymer Processing, Urumqi 830052, China

**Keywords:** hexamethylenetetramine, molecular rotation, semiconductors, reversible dielectric properties, ferromagnets

## Abstract

Multifunctional coupled hybrid materials have extremely high potential for application in a variety of complex scenarios owing to advantages such as versatility and controllable properties. In this study, a novel functional material with electromagnetic coupling properties [Ni(NCS)_4_(C_6_H_13_N_4_)_2_] (**1**) was obtained by naturally evaporating an aqueous solution of nickel chloride hexahydrate, hexamethylenetetramine (HMTA), and potassium thiocyanate as raw materials. Structure–property characterization revealed that **1** crystallized in the *P*2_1_/n space group with a two-dimensional (2D) network structure formed by hydrogen-bonding interactions between neighboring nickel complexes. Calculations using the Gaussian program indicated that HMTA exhibited pronounced spatial molecular rotation. This induced obvious reversible dielectric cycling near 240 K, giving rise to semiconducting properties and an optical band gap of 3.35 eV. Molecular rotation caused changes in the 2D network structure, inducing short-range magnetic ordering in the temperature range of 2–50 K. This resulted in the formation of a potential ferromagnet and the presence of a distinct reversible redox peak in the −0.2–0.8 V potential range. Structure–property analyses showed that **1** is a supramolecular rotation-induced semiconducting multifunctional crystalline material with reversible electromagnetic coupling properties.

## 1. Introduction

With the rapid development of science, technology, and society in recent years, organic–inorganic multifunctional hybrid materials have gradually gained popularity as smart materials [1,2,3,4,5,6,7,8,9]. These novel materials, which combine the advantages of inorganic and organic materials, have demonstrated superior optical, electrical, and magnetic properties. However, not all hybrid materials are capable of exhibiting ideal functional properties [10,11,12,13,14,15]. The structural design of organic and inorganic components, modulation of components, and relationships between properties have been the focus of pertinent research to date [16,17,18,19,20,21,22,23,24,25,26,27]. Nitrogen-containing compounds are an important class of organic compounds in the construction of organic–inorganic hybrid phase-change materials. Their abundance of structural types and diverse properties have allowed for the design and synthesis of novel nitrogen-containing metal–organic complex-based multifunctional materials [28,29,30,31,32,33,34,35,36,37,38,39]. In particular, multifunctional materials that combine pertinent physicochemical properties, such as optical, electrical, magnetic, and thermal properties, have immense application prospects in energy transfer and conversion, data storage, smart switches, information transfer, and other fields related to interaction technology [40,41,42,43,44,45,46].

Hexamethylenetetramine (HMTA), also known as urotropine, is a nitrogen-containing heterocyclic compound with a highly symmetrical, rigid stereostructure. The low cost and low toxicity of this tetramine make it suitable for use as an environmentally friendly organic ligand. It readily forms cage-like structures and can adopt coordination modes ranging from terminal monodentate to tetradentate [47]. Compared with other nitrogen-containing organic compounds, HMTA possesses attractive features such as a highly symmetrical molecular scaffold and four equivalent nitrogen atoms that act as hydrogen-bond acceptors. Yao et al. [20] reported two two-dimensional (2D) Hoffman-type compounds, {Fe(3,4-bpt)_2_[M(CN)_4_]} (3,4-bpt = 3,4-bis(4-pyridyl)thiophene; M = Pt (**2**), Pd (**3**)), which showed reversible switching in magnetism, dielectricity, and thermochromism actuated by the spin transition of Fe(II). X-ray structural analyses revealed that variations in the Fe(II) coordination sphere caused changes in the local electric dipoles, leading to asymmetry between the high-temperature and low-temperature phases and resulting in dielectric excitation. Magnetic susceptibility analyses indicated that **2** and **3** experienced thermo-induced spin-state transitions at 20 K, with consistent dielectric and magnetic properties. Both **2** and **3** exhibited a well-defined bistable state within the temperature range of 100–220 K. They also exhibited high sensitivity to external light irradiation and could be reversibly switched between the HS and LS states under alternating laser irradiation at 532 and 808 nm. This study provided a new platform for spin crossover-actuated multichannel switches and offered valuable insights into the design and synthesis of novel optical, electrical, and magnetic materials with superior properties. Xu et al. [48] reported a novel ferroelastic semiconducting hybrid organic–inorganic perovskite (C_3_H_7_N_2_S)PbBr_3_ with a ferroelastic phase transition at 395 K and an optical band gap of 3.43 eV. The compound had a one-dimensional (1D) BaNiO_3_-type structure and underwent a dielectric switch near the phase-transition temperature. This was the first report of a ferroelastic semiconductor material. Successful preparation of this material indicates valuable development potential for functional materials with semiconducting properties and excellent dielectric properties. On this basis, we focused on the structural and functional design of organic–inorganic hybrid materials in this study. Nickel chloride hexahydrate (NiCl_2_·6H_2_O), HMTA, and potassium thiocyanate (KSCN) were used as raw materials for a self-assembly reaction and a novel organic–inorganic hybrid nickel complex [Ni(NCS)_4_(C_6_H_13_N_4_)_2_] (**1**) was obtained using the solvent evaporation method. The structural, thermal, magnetic, and electrical properties of the obtained crystals were analyzed using single-crystal X-ray diffraction (XRD), variable-temperature infrared (IR) spectroscopy, powder XRD, thermogravimetric analysis (TGA), Hirshfeld surface analysis, and cyclic voltammetry (CV). The potential energy of rotation, semiconducting properties, dielectricity, and magnetism were also studied. The results indicated that the coordination of spherical organic cations with the inorganic framework synergistically affected the microstructure, thus promoting the transformation of multiple physical properties. The utilization of molecular rotation to couple the reversible cyclic dielectric, ferromagnetic, and semiconducting properties facilitates the expansion of research on hybrid materials and the development of integrated dielectric–optoelectronic devices.

## 2. Results and Discussion

### 2.1. Variable-Temperature IR Spectra of Compound ***1***

**Figure 1:**
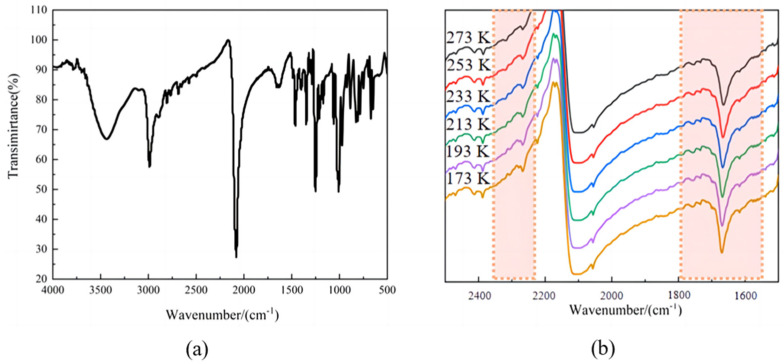
IR spectrum (**a**,**b**) variable-temperature IR spectra of compound **1.** The shadow represents the part of the peak change within a certain interval.

The main components of **1** were preliminarily determined using IR spectroscopy. Compound **1** was ground or crushed with an appropriate proportion of KBr prior to the analysis. The IR spectra of **1** within the range of 4000–500 cm^−1^ revealed an absorption peak near 1000 cm^−1^, which was attributed to the stretching vibrations of C–N in HMTA (Figure S2a). The characteristic peak at 2078 cm^−1^ overlapped with the stretching vibrations of C≡N and asymmetric stretching vibrations of N=C=S in KSCN (Figure S2b). Based on the characteristic peaks in Figure 1a, we preliminarily deduced that compound **1** was the target product and contained HMTA and isothiocyanate. To further determine the relationship between crystal structure and temperature, the IR spectra of compound **1** were acquired at 273, 253, 233, 213, 193, and 173 K. Using KBr as the background, the samples were mixed with KBr powder and pressed. Then, under the temperature control condition of liquid nitrogen, the temperature was cooled from 273 K to 173 K at a rate of 2 K/min for a variable temperature test. As shown in Figure 1b, the number of characteristic peaks of the N=C=S group in the wavenumber range of 2200–2300 cm^−1^ gradually increased when the temperature was raised to 233 K. Within the range of 1500–1800 cm^−1^, the peak attributed to the stretching vibrations of C–N in HMTA became increasingly thinner and sharper. Therefore, it can be deduced that coordination bond strength in **1** increased progressively with a decrease in temperature.

### 2.2. Single-Crystal X-Ray Structures of Compound ***1***

**Figure 2:**
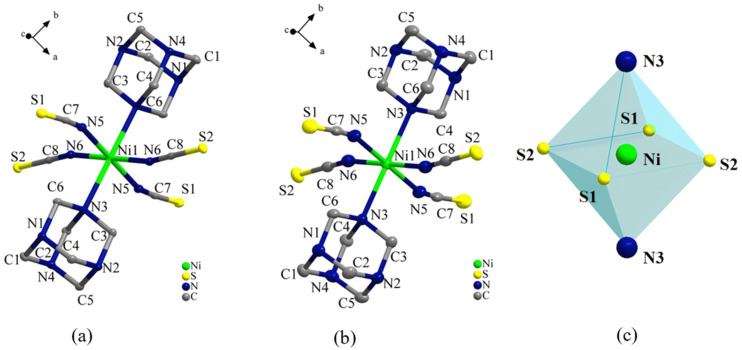
Asymmetric unit of compound **1** at (**a**) 100 K and (**b**) 293 K; (**c**) octahedral configuration.

Figure 2a,b show the asymmetric unit of **1** at low temperature (100 K) and room temperature (293 K), respectively. The asymmetric unit consisted of two protonated HMTA molecules with top–bottom symmetry and four isothiocyanate molecules symmetrically arranged within a plane, coordinated to a central nickel ion. A discrete octahedral nickel complex [Ni(NCS)_4_(C_6_H_13_N_4_)_2_] (**1**) was ultimately formed. Forces between molecules within the same cell contributed to the formation of a zero-dimensional (0D) structure through coordination bonding.

**Figure 3:**
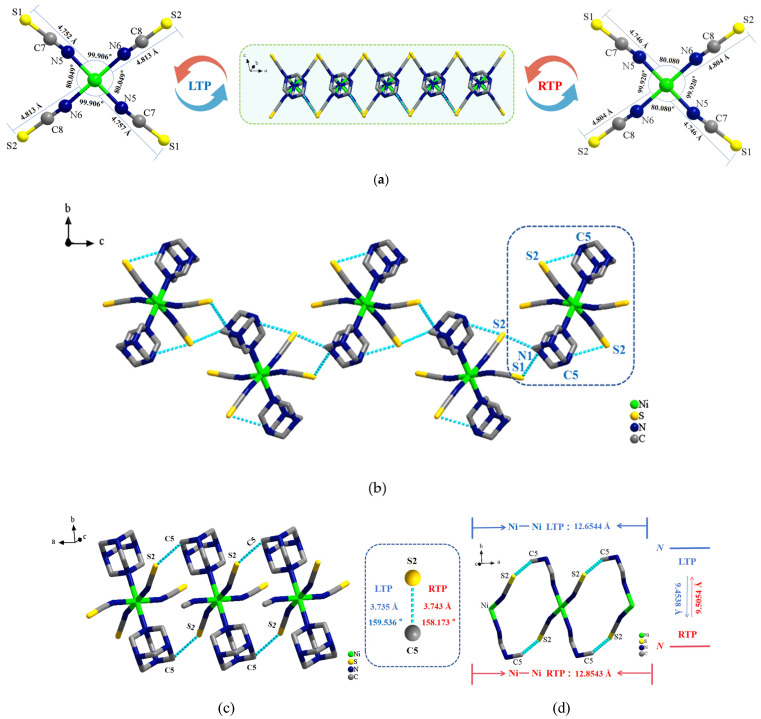
(**a**) Inorganic anionic structure of compound **1** in the *ac* plane: (**b**) 1D supermolecular chain in the *bc* plane; (**c**) 1D supermolecular chain in the *ab* plane; and (**d**) hydrogen-bonded looped backbone chain.

Figure 3a shows the structure of the (**1**) in the *ac* plane. At low and room temperatures, slight changes occurred in the range of distances between the central nickel atom and sulfur atoms at the tetrahedral vertices, as well as in the bond lengths and angles, as shown in Table S1. Neighboring nickel complexes in **1** were linked by the N1–H1···S1 and N1–H1···S2 hydrogen bonds and extended indefinitely along the *c*-axis, forming the 1D hydrogen-bonded supermolecular chain structure shown in Figure 3b. Within the *ab* plane, S2 in an isothiocyanato-nickel complex was linked to C5 in the neighboring HMTA molecule through the C5-H5B···S2 intermolecular hydrogen bond. Extension along the *a*-axis led to the formation of a 1D hydrogen-bonded supermolecular chain structure, as shown in Figure 3c. Three adjacent centrally coordinated nickel atoms in the *ab* plane were selected to form the hydrogen-bonded looped backbone supermolecular chain shown in Figure 3d. With an increase in temperature, the distance between the three adjacent centrally coordinated nickel atoms (Ni···Ni) increased from 12.654 Å at low temperature to 12.854 Å at room temperature. The lengths and angles of the hydrogen bonds also changed significantly, as shown in Table S2. A comprehensive analysis revealed that the bond lengths and angles of the isothiocyanato-nickel complexes changed significantly with a change in temperature. This led to distortion of the hexacoordinated octahedral structure, providing effective spaces for molecular rotation and libration of HMTA cations around the N–Ni axis.

**Figure 4:**
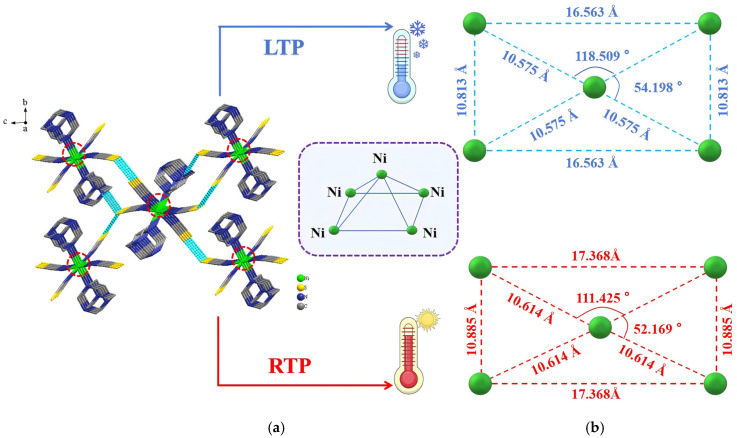
(**a**) 2D supramolecular stacked network of Compound **1** in the *bc* plane; (**b**) Spatial structure. The red circles represent the key symbols, and the atoms within the circles are Ni atoms.

As shown in Figure 4a, compound **1** exhibited a 2D supramolecular hydrogen-bonded network in the *bc* plane that was constructed by N1–H1···S1, N1–H1···S2, and C5–H5B···S2 interactions. Repetitive and orderly stacking of the front-back neighboring supramolecular hydrogen-bonded networks along the *a*-axis was also observed (Figure S3). To further investigate the influence of metal backbone distance on the frame structure, five nickel atoms were selected to form a square pyramid, and the changes in pyramid edge length, lengths of the four pyramid base sides, and angles formed with the apex were measured (Figure 4b). The results indicated that the pyramid base length, width, and pyramid edge length changed with temperature. Measurements of the angles of the framework along the *c*-axis and *a*-axis revealed varying extents of change. The change in temperature caused the angle between nickel atoms along the *c*-axis to decrease from 54.198° at the low temperature to 52.169° at room temperature. The angle between nickel atoms along the *a*-axis also decreased from 118.509° at the low temperature to 111.425° at room temperature. This demonstrated that the supramolecular network framework of **1** contracted along the *c*-axis under the influence of thermal molecular motion. Such a change may have distorted the octahedral structure framework of **1**, making it more likely for the physical properties of the material to change.

### 2.3. XRD Spectrum of Compound ***1***

**Figure 5:**
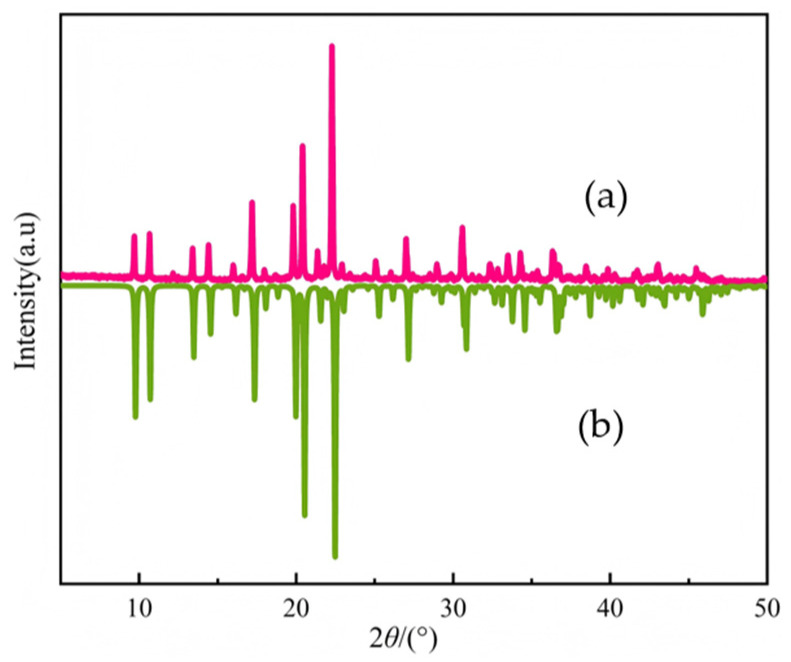
(**a**) Powder X-ray diffraction (**b**) simulated Powder X-ray diffraction of compound **1**.

Dry, pure, and transparent compound **1** samples were selected for powder XRD; mercury was used to simulate the crystal structure of the CIF file, and Figure 5 was obtained. The experimental PXRD pattern (Figure 5a), which featured distinct, characteristic peaks, and simulated PXRD pattern deduced from the simulated single-crystal structure (Figure 5b) indicated good sample fitting, with the relative intensity of peaks perfectly matching the calculated peak positions. The comparison of experimental and simulated data further confirmed that **1** was a single-phase, pure substance, which laid the foundation for subsequent testing of the physical properties of the material.

### 2.4. Thermal Analysis of Compound ***1***

**Figure 6:**
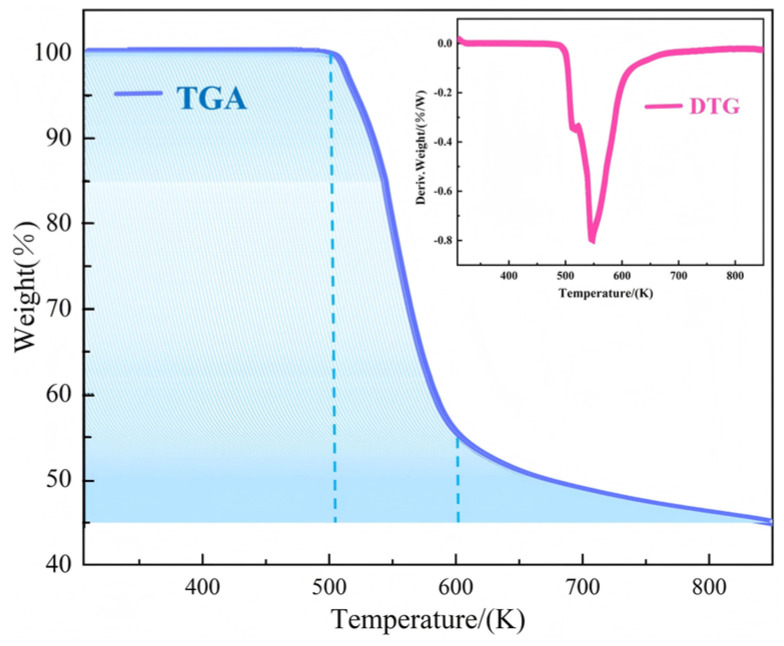
TGA and differential thermogravimetry.

The thermal stability of **1** was tested by selecting an appropriate amount of dry sample for TGA analysis within the temperature range of 300–850 K; the heating rate was 10 K/min. Measured data were used for plotting the TGA and differential thermogravimetry (DTG) curves shown in Figure 6. An analysis of the curves indicated that the thermal stability of **1** could be roughly divided into three stages. In the first stage (300–500 K), compound **1** did not lose any mass. In the second stage (500–600 K), the mass loss rate was 44%, which was basically consistent with the theoretical mass loss rate of 40% for tetracoordinated isothiocyanate ions. Concurrently, a strong endothermic peak appeared in the DTG curve. The remaining mass occurred in the third stage (600–850 K). In this stage, the mass loss rate of **1** was 12%, which closely matched the theoretical loss for half of the HMTA (14%). The remaining stable residue was presumed to result from incomplete decomposition of the remaining HMTA cations and nickel compound. The TGA results were consistent with crystal structure data for **1**, indicating that the crystalline material possessed good thermal stability.

### 2.5. Hirshfeld Surface Analysis of Compound ***1***

**Figure 7:**
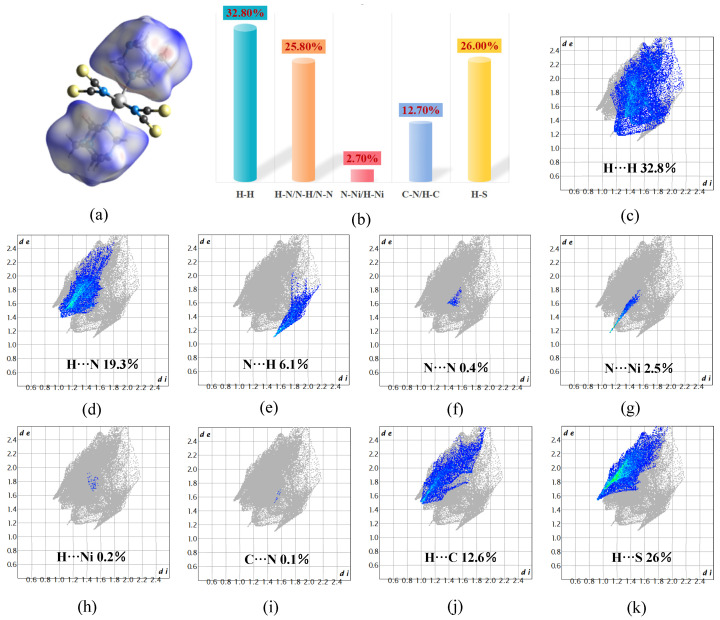
(**a**) Hirshfeld surface of compound **1**: (**b**) bar chart; (**c**–**k**) 2D fingerprint plots generated with the *d_norm_* parameter.

The interactions between atoms in the crystal structure were calculated and analyzed to determine the structural variability of the interactions between various molecules. Atoms comprising HMTA and the isothiocyanato-nickel complexes were selected for model construction. Subsequently, the Hirshfeld surface was imaged along 2D fingerprint plots generated using the normalized contact distance between atoms (*d_norm_*) as a parameter, thereby providing an overview of the interactions between different atoms. As shown in Figure 7a, two HMTA molecules were included in the Hirshfield surface. Individual interactions were quantified by the fingerprint plots shown in Figure 7c–k: H···H interactions accounted for 33.8% of all interactions in the crystal structure; H···N, N···H, and N···N interactions for 25.8%; H···S interactions for 26%; H···C and C···N interactions for 12.7%; and N···Ni and H···Ni interactions for 2.7%. These findings indicate the presence of strong hydrogen-bonding interactions between the molecules of **1**. This is consistent with the results of the crystal structure analysis, which indicated the presence of 2D hydrogen-bonded network structures within the complexes.

### 2.6. Potential Energy of Cation Rotation in Compound ***1***

**Figure 8:**
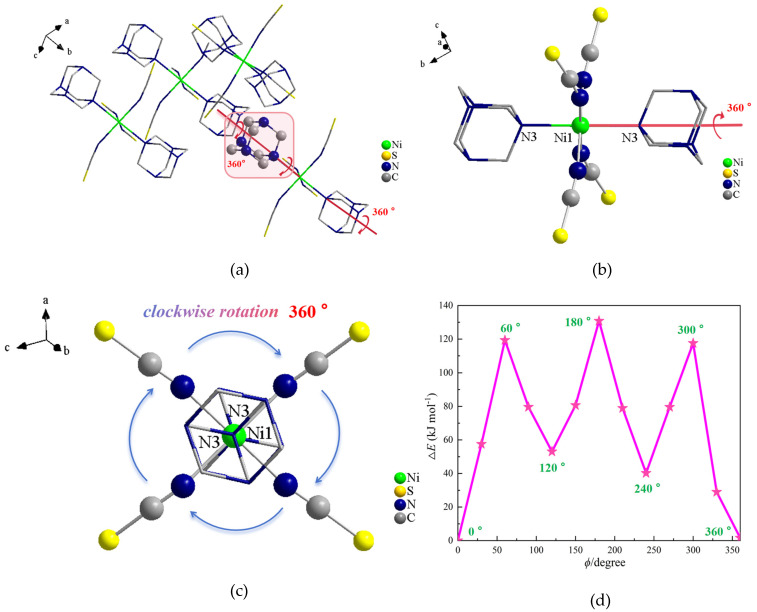
(**a**–**c**) Model of HMTA rotation around the N–Ni axis; (**d**) Potential energy curve of rotation.

To determine whether the molecular motion of the HMTA cations and tetrakis(isothiocyanato)-nickel complexes contributed significantly to the structure and physical properties of the compound, the potential energy of rotation of **1** was evaluated using the RHF/9-31(d) method in the Gaussian program. In the present paper, a model containing three adjacent tetrakis(isothiocyanato)-nickel complexes was constructed (Figure 8a), and the function for the rotation angle (*ϕ*) of the HMTA molecules around the N–Ni axis was established using 30° as a rotational unit (Figure 8b,c). Figure 8d shows the potential energy curve of rotation formed by molecular rotation of the HMTA molecules around the N–Ni axis. This reflects the dependence of the relative energy (Δ*E*) on the angle of rotation, which was set to zero when *ϕ* = 0°. Three potential energy minima could be observed at *ϕ* = 0°, 120°, and 240°, indicating that HMTA molecules were prone to a 120° flip-flop motion under certain external conditions. The local maxima of the HMTA molecules occurred at *ϕ* = 60°, 180°, and 300°, with energies of 119.90, 131.02, and 117.91 kJ mol^−1^, respectively. These values were much lower than that reported by Akutagawa et al. [49] (250 kJ mol^−1^), indicating that HMTA molecules can undergo a 360° flip-flop motion within certain spaces. However, the results of the present study were consistent with the maximum, minimum, and average rotational potential energies reported by Shi et al. [50], which validates the three minima and three maxima exhibited by the HMTA molecules. This indicates that within the crystal space, changes in external conditions such as temperature and electric field can induce the rotation of HMTA molecules within the microstructure, thereby leading to abrupt changes in the dielectric, ferroelectric, magnetic, and optical properties of the material. Ultimately, a novel class of 2D hydrogen-bonded crystalline materials with stator–rotor coordination complexes was obtained.

### 2.7. Semiconducting Properties of Compound ***1***

**Figure 9:**
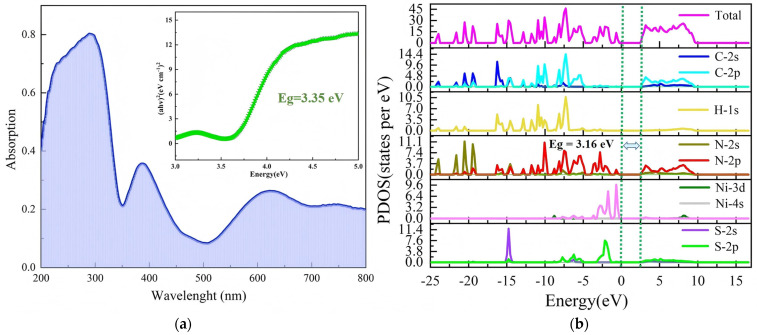
(**a**) UV–Vis absorption spectrum and (**b**) density of states (PDOS) plots of compound **1**.

Organic–inorganic hybrid materials may potentially serve as novel semiconducting materials. We acquired the solid-state UV-diffuse reflectance spectrum of **1** at room temperature to investigate its semiconducting properties, with measurements performed in the 200–800 nm range. The absorption onset shift was located around 300 nm, and the absorption curve showed a distinct band–edge cutoff. No absorption tail or exciton features and barely any defects were observed, indicating that the material consisted of high-quality functional crystals [15,51]. As shown in Figure 9a, compound **1** exhibited different electron-transition behaviors, with UV–Vis absorption peaks appearing at 290, 388, and 620 nm. An analysis revealed that absorption peaks within the range of 250–280 nm were attributed to π→π* electron transitions of the conjugated structure in HMTA and σ→π* transitions of NCS^−^. In material structures, the presence of amino groups interferes with and affects the electronic properties of molecules. The HMTA absorption peak at 350 nm is typically associated with the conjugated structure [52,53,54]. The broad absorption peak near 620 nm corresponded to intermolecular interactions in [Ni(NCS)_4_]. To determine whether **1** was a semiconductor, the energy band structure of its model was calculated using DFT. By characterizing the structure of **1** using the UV–Vis spectrum, the calculated band gap (Eg) was determined to be ≈3.35 eV (Figure 9a). This is slightly smaller than the band gap of 3.43 V reported by Xu et al. as mentioned in the introduction. Therefore, we preliminarily deemed **1** to be semiconducting material. The band gap of **1** may have resulted from slight coordination distortion within the octahedral structure formed by the isothiocyanato-nickel complexes. To investigate the band gap distribution of **1** in greater depth, we calculated the partial density of states (PDOS) of the material, which reflects the electron distribution across the various orbitals. Through a combined analysis of the total density of states (TDOS) plot, PDOS plots, and band structures, the contributions of different atoms to the conduction and valence bands can be determined. As shown in Figure 9b, the valence band PDOS of **1** under normal pressure was within the range of −15.61 to −14.202 eV and was primarily composed of the following electronic states: S 2s, N 2s2p, H 1s, C 2s2p, and a small amount of S 2p. The conduction band minimum and valence band maximum were both located in the same region of the Brillouin zone, which further confirmed the existence of an indirect band gap semiconductor. A theoretical band gap value of 3.16 eV was calculated, which slightly differed from the experimentally determined value of 3.35 eV owing to the limitations of DFT. Within the energy range, the C-2s2p orbitals almost completely overlapped with the H-1s and N-2s2p orbitals, indicating strong covalent interactions among the inorganic cations. The 4s orbital of the Ni atoms mainly occupied the energy band at the top of the valence band, whereas the energy band at the bottom of the conduction band was contributed by the 2p orbital of S atoms. The electronic states of the Ni and S atoms mainly contributed to the conduction band minimum and valence band maximum. This indicated that the semiconductor band gap of the material was dependent on the inorganic coordination backbone, which was indicative of good semiconductor properties.

### 2.8. Dielectric Properties of Compound ***1***

**Figure 10:**
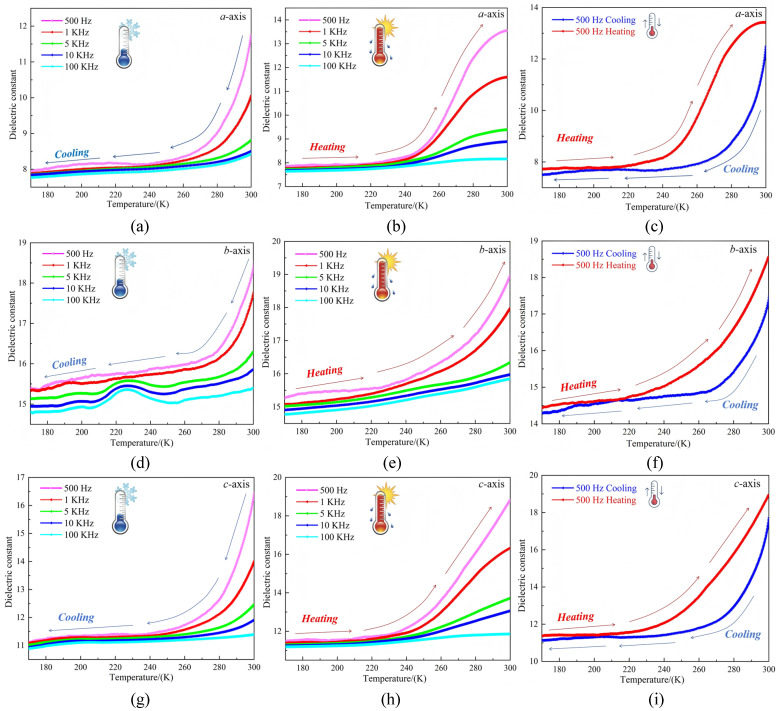
(**a**–**c**) Cyclic dielectric constant curves of **1** along the *a*-axis during cooling, heating, and at 500 Hz; (**d**–**f**) Curves along the *b*-axis during cooling, heating, and at 500 Hz; (**g**–**i**) Curves along the *c*-axis during cooling, heating, and at 500 Hz.

Dielectric properties such as frequency, temperature, and surface roughness are vital to guiding material testing [55]. Samples of **1** with good crystal shapes were selected, and the three axial directions (*a*, *b*, and *c*) were identified. Capacitors were constructed from the samples using conductive silver paste and copper wires, and dielectric testing was carried out in the frequency range of 500–100 kHz. Figure 10 shows that along the *a*-axis, the dielectric constant at varying frequencies decreased slowly within the temperature range of 170–310 K during the cooling process (Figure 10a). When the temperature was ≈240 K, **1** exhibited a dielectric anomaly, and the dielectric constant did not change further upon cooling beyond 240 K. During the heating process from 180 to 300 K (Figure 10b), a dielectric anomaly was produced at 240 K, with the dielectric constant increasing sharply beyond 240 K. The cyclic dielectric constant curves at the other frequencies shown in Figure 10c and Figure S4 clearly indicated that **1** underwent reversible dielectric anomalous cycling along the *a*-axis. In the direction of the *b*-axis, the dielectric constant at varying frequencies decreased slowly within the temperature range of 170–310 K during the cooling process (Figure 10d). When the temperature was ≈225 K, a dielectric anomaly was exhibited by **1** at frequencies of 5, 10, and 100 kHz. This was followed by a gradual decrease beyond 210 K. During the heating process from 170–300 K (Figure 10e), compound **1** showed a dielectric anomaly at 240 K, with the dielectric constant increasing sharply when the temperature exceeded 240 K. The cyclic dielectric constant curves at the other frequencies shown in Figure 10f and Figure S5 indicated that **1** exhibited complete dielectric reversibility along the *b*-axis. In the direction of the *c*-axis, the dielectric constant at the various frequencies decreased gradually within the temperature range of 170–310 K during the cooling process (Figure 10g). When the temperature was ≈240 K, **1** exhibited a dielectric anomaly, and the dielectric constant did not change further upon cooling beyond 240 K. During the heating process from 170–300 K (Figure 10h), **1** showed a dielectric anomaly at 240 K. The dielectric constant increased sharply when the temperature was increased beyond 240 K, thereby forming a step-like pattern similar to that of the dielectric constant trend along the *a*-axis. As shown by the cyclic dielectric constant curves at the other frequencies (Figure 10i and Figure S6), **1** underwent complete reversible dielectric cyclic curves along the *c*-axis. During the cooling and heating processes, the dielectric constant of **1** was positively correlated with frequency. This was consistent with the variable-temperature ranges generated in the variable-temperature IR spectroscopic analysis of **1** (Figure 10b). Structural analysis revealed that the distinct reversible dielectric anomaly was primarily caused by the molecular rotation of HMTA in **1**, which induced the expansion and contraction of bond lengths and angles within the structure and changes in the 2D hydrogen-bonded network framework. The resultant structural deformation led to good cyclic behavior of the dielectric properties of the crystalline material.

### 2.9. Magnetic Testing of Compound ***1***

**Figure 11:**
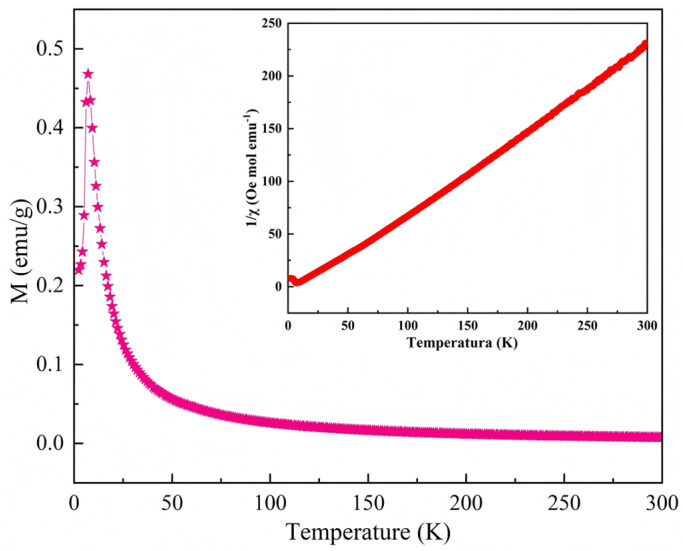
M–T curve of compound **1**.

The presence or absence of magnetic properties in **1** was determined by measuring magnetization (M) as a function of temperature (T) in the range of 2–300 K under an applied magnetic field of 1000 Oe. As shown by the temperature-dependent magnetization (M–T) curve in Figure 11, short-range ferromagnetic ordering may have existed between 2 and 50 K, whereas paramagnetic behavior was observed in the 50–300 K range. The Curie temperature, *T*_c_, was determined to be 7.63 K by differential derivation (Figure S7). No obvious hump appeared in the curve for **1** within the temperature range of 50–300 K. This is characteristic of linear antiferromagnetic interactions and indicates that intra-chain coupling was inherently magnetic [56]. The M–T curve showed a gradual increasing trend with a decrease in temperature. Within the low-temperature range, magnetization increased rapidly until the temperature reached 6 K. The magnetization curve exhibited a clear turning point, which provided a preliminary indication of the ferromagnetic nature of **1**. The calculated values for the parameters *C* and *Θ* were −0.1002 cm^−3^ mol and 0.079 K, respectively (Figure 11). The positive value of *Θ* further confirmed that the spontaneous magnetization of **1** would be enhanced, indicating obvious ferromagnetic characteristics [57]. Compound **1** followed the ideal Curie–Weiss behavior at temperatures above 50 K. The calculated effective magnetic moment, *μ*_eff_, was 2.51 *μ*_B_, which was close to the theoretical magnetic moment of nickel (2.83 *μ*_B_). This indicates that the magnetic properties of **1** were primarily contributed by the nickel ions. The calculated *μ*_eff_ was higher than that reported by Uher et al. (2.00 *μ*_B_) [58], demonstrating that **1** was a ferromagnetic crystalline material with immense application potential.

### 2.10. Cyclic Voltammetry (CV) of Compound ***1***

**Figure 12:**
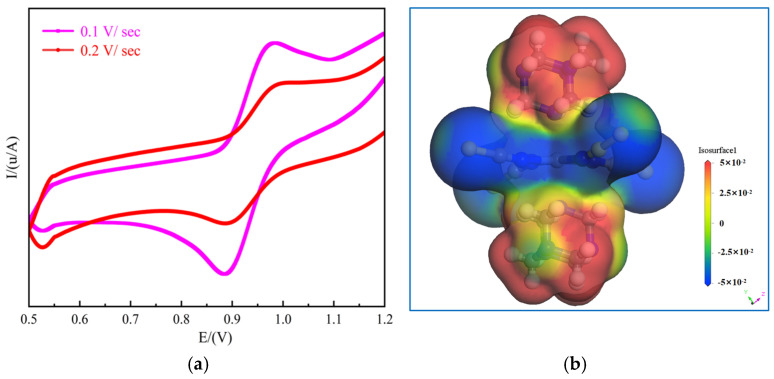
(**a**) Cyclic voltammogram and (**b**) MEP map of compound **1**.

The electrochemical properties of **1** were investigated by CV in a three-electrode system (glass carbon electrode, auxiliary electrode, and reference electrode) was tested in a mixture of 0.1 mol/L H_2_SO_4_ and 0.5 mol/L Na_2_SO_4_. Figure 12a shows the cyclic voltammogram of **1**, which reveals a distinct pair of reversible redox peaks within the potential range of –0.2 to 0.8 V. The half-wave potential, *E*_1/2_, was 0.418 V at a scan rate of 0.1 V/s and 0.419 V at a scan rate of 0.2 V/s. Therefore, the redox peaks could be determined using the equation *E*_1/2_ = (*E*pa + *E*pc)/2, where *E*pa is the anodic peak potential and *E*pc is the cathodic peak potential. The redox peaks were consistent with the electron transfer of Ni^2+^/Ni in the nickel complexes, with the electron-transfer process during electrolysis being reversible. Wang et al. [59] performed CV by using NiCl(OH)NS/CC as a working electrode for electrochemical sensing under alkaline conditions. At a scan rate of 50 mV/s, NiCl(OH)NS/CC exhibited a pair of reversible anodic and cathodic peak potentials corresponding to the Ni^3+^/Ni^2+^ process, implying that peak separation increased with an increase in scan rate. This is similar to the electrochemical properties of [Ni(NCS)_4_(C_6_H_13_N_4_)_2_] synthesized in the present study, which indicates that **1** possessed good cycling stability and electrochemical properties. To investigate the electrostatic interactions between the HMTA cations and isothiocyanato-nickel complex anions, the molecular electrostatic potential (MEP) was calculated using the GGA-PBE functional, which was related to molecular reactivity and intermolecular forces [60]. Figure 12b shows the electrophilic and nucleophilic interactions between the protonated HMTA molecules and isothiocyanato-nickel complex anions. MEP was plotted on an isosurface within the range of 5.000 eV (red region) to −5.000 eV (blue region), where red indicates stronger intermolecular attractions. Our results indicated that electrophilic reactivity was primarily concentrated on the electronegative nitrogen atoms and hydrogen atoms in the HMTA cations. The blue region represents the area of maximum repulsive potential. Owing to the partial shielding of the nuclear charge density, nucleophilic reactivity was mainly limited to the carbon atoms and sulfur atoms within the isothiocyanato-nickel complex anion structure. A comprehensive analysis revealed that the nucleophilic and electrophilic reactions influenced the final reaction rate, conversion efficiency, and kinetic properties in the electrochemical reactions.

## 3. Materials and Methods

### 3.1. Main Reagents and Instruments

The following reagents were used: nickel chloride hexahydrate (chemically pure grade; Jinjinle Chemical Co., Ltd., Shanghai, China), potassium thiocyanide (chemically pure grade; Jinjinle Chemical Co., Ltd., Shanghai, China), HMTA (chemically pure grade; Hutubi County Ruiyuantong Chemical Co., Ltd., Xinjiang Uyghur Autonomous Region, China), and hydrochloric acid (analytically pure grade; Tianjin Yongsheng Fine Chemical Co., Ltd., Tianjin, China). The instruments and software used in this study included an FTIR8700 IR spectrometer (Shimadzu, Kyoto, Japan) and Bruker Smart Apex II single-crystal X-ray diffractometer (Rigaku, Tokyo, Japan) equipped with a graphite monochromatic Mo-Kα radiation source (λ = 0.07103 Å). Single-crystal samples measuring approximately 0.13 × 0.12 × 0.11 mm without cracks and with good transparency were selected for XRD analysis. Diffraction data for **1** at a low temperature (100 K) and room temperature (293 K) were collected within a certain angle range, and diffraction points were selected for direct analysis of compound structures in the SHELXS-97 software [61,62]. Anisotropic temperature factors and the coordinates of non-hydrogen atoms were structurally corrected using the full-matrix least-squares method. A powder X-ray diffractometer (Bruker, Berlin, Germany), Q50 thermogravimetric analyzer (TA Instruments, New Castle, DE, USA), and Hirshfeld surface analysis (using the CrystalExplorer program, 608bb32) were also used for sample characterization. In CrystalExplorer, density functional theory (DFT) was used to calculate the interactions between atoms in crystal structures [63,64,65]. Using the Gaussian program (G9M016773358589W-5044N), molecular rotation was calculated using the RHF/9-31(d) basis set, and the relative structural energy was computed using a semi-empirical method. A UV-3600 UV-diffuse reflectance spectrometer (Shimadzu, Japan), physical property measurement system (Quantum Design, San Diego, CA, USA), and TH2828 LCR meter (Changzhou Tonghui Electronic Co., Ltd., Changzhou, China) for dielectric constant measurement, and CHI700E electrochemical workstation (Shanghai Chenhua Instrument Co., Ltd., Shanghai, China) were also used.

### 3.2. Synthesis Methods

**Figure 13:**
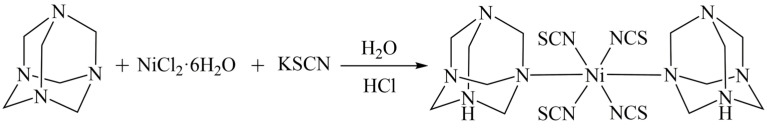
Synthesis of compound **1**.

The results of varying the mixing ratios of the three raw materials indicated that 1:6:2 was the optimal ratio for crystal growth. Based on the predetermined ratio, 0.2 g (0.84 mmol) of NiCl_2_·6H_2_O, 0.5 g (5.14 mmol) of KSCN, and 0.2 g (1.42 mmol) of HMTA were separately dissolved in 5 mL of aqueous solution. Afterward, 0.25 mL of hydrochloric acid (HCl) was added dropwise with continuous stirring to the solvent containing NiCl_2_·6H_2_O, and the solution was left to stand for 10 min. The aqueous solutions containing KSCN and HMTA were mixed, and the mixed solution was added dropwise to the HCl-containing NiCl_2_·6H_2_O solution. Subsequently, the resultant solution was left to stand in a cool place to allow natural solvent evaporation, and light blue crystals were obtained after 7 d (Figure S1). Figure 13 shows the synthesis reaction. Compound **1** consists of a nickel ion, four isothiocyano groups, and two coordinated molecules of hexamethylenetetramine.

### 3.3. Measurement of Crystal Structure of Compound ***1***

Table 1 shows that at temperatures of 100 and 293 K, compound **1** had the empirical formula C_16_H_26_N_12_NiS_4_, a relative molecular mass of 573.44, and crystallized in the *P*2_1_/n space group of the triclinic system.

## 4. Conclusions

A novel organic–inorganic hybrid nickel complex [Ni(NCS)_4_(C_6_H_13_N_4_)_2_] (**1**) was obtained by naturally evaporating an aqueous solution of nickel chloride hexahydrate, HMTA, and potassium thiocyanate. The single-crystal XRD patterns indicated that **1** crystallized in the *P*2_1_/n space group. Within the unit cell, the octahedral nickel complex formed by four molecules of isothiocyanate and two molecules of protonated HMTA was connected by coordination bonds to form a 0D structure. The neighboring complexes were linked by hydrogen bonds to form a 2D network structure. Temperature-induced changes in the lengths and angles of hydrogen bonds formed among the various molecules caused slight distortions in the octahedron within the spatial structure of **1**. This provided spaces for the molecular rotation of HMTA. At varying electric field scan rates, **1** exhibited obvious reversible redox peaks, while MEP calculations revealed strong, attractive forces within the structural framework. The potential energy curve of the HMTA molecules exhibited three symmetric peaks, with a maximum rotational energy of 131.02 kJ mol^−1^; this is much lower than the theoretical rotational energy range, allowing for the generation of a favorable 120° flip-flop motion. Consequently, reversible dielectric properties were induced in **1** at 240 K, leading to a calculated theoretical band gap of 3.16 eV, which clearly indicates semiconducting behavior. The rotation of HMTA molecules and deformation of the inorganic backbone led to short-range magnetic ordering in the material within the temperature range of 2–50 K, which induced pronounced ferromagnetic properties. Our results demonstrate that the HMTA molecular rotation-induced deformation of the structural framework of **1** contributes to the development of a novel multifunctional crystalline material with reversible electromagnetic coupling and semiconducting properties.

## Figures and Tables

**Table 1:** Crystallographic data of compound **1**.

Temperature	100 K	293 K
Empirical formula	C_16_H_26_N_12_NiS_4_	C_16_H_26_N_12_NiS_4_
Formula weight	573.44	573.44
Crystal system	Monoclinic	Monoclinic
Space group	*P*2_1_/n	*P*2_1_/n
*a*/Å	6.3767(3)	6.4272(6)
*b*/Å	10.8130(5)	10.8858(13)
*c*/Å	16.5638(9)	16.6018(16)
*α*/°	90	90
*β*/°	94.187(5)	94.103(9)
*γ*/°	90	90
Volume/Å^3^	1139.04(10)	1158.6(2)
Z	2	2
ρ_calc_g/cm^3^	1.672	1.644
*μ*/mm^−1^	1.253	1.231
*F*(000)	596.0	596.0
Crystal size/mm^3^	0.14 × 0.13 × 0.12	0.14 × 0.13 × 0.12
Measured 2θ range (°)	4.502–49.978	4.478–49.998
*R*_int_	0.0262	0.0319
GOF	1.030	1.125
CCDC	2,370,103	2,370,104

## Data Availability

The concern of corresponding author data can be provided.

## Supplementary Materials

The following supporting information can be downloaded at: https://www.mdpi.com/article/10.3390/ijms26094050/s1.
