# Supplementary material for: Rotation of Hexamethylenetetramine Molecules Induces Reversible Electromagnetic Coupling Properties in Isothiocyanato-Nickel Complexes
Source: Int J Mol Sci. 2025 Apr 25;26(9):4050. doi: 10.3390/ijms26094050 (PMC12071420; doi:10.3390/ijms26094050)
Supplement: Supplementary file 1 [file ijms-26-04050-s001.zip › ijms-3566596-supplementary.pdf]

Rotation of hexamethylenetetramine molecules induces reversible electromagnetic coupling properties in isothiocyanato-nickel complexes

Adila • Abuduheni[1], Leilei Zhou[1], Yubing Yao[2], Yang Liu[*1,3], Hongzhi Hu[1,3], Zunqi Liu[*1,3]
(1. Chemistry and Chemical Engineering College Xinjiang Agricultural University;
2. School of Computer and Information Engineering，Xinjiang Agricultural University,
3. Xinjiang Sub-Center National Engineering Research Center of Novel Equipment for Polymer Processing，Urumqi 830052, Xinjiang;)

Table S1. The bond length and angle of compound **1**

Chemical Bond	Bond Length /Å	$\lvert\delta = \bar{x} - \mu\rvert$%	Chemical Bond	Bond Length /Å	$\lvert\delta = \bar{x} - \mu\rvert$%
			100 K		
Ni1-N5	2.051(2)	10.76%	N3-Ni1-N5	92.08	43.40%
Ni1-N6	2.038(2)	12.06%	N3-Ni1-N3	180.00	44.52%
S1-C7	1.647(3)	51.16%	N3-Ni1-N6	88.75	46.73%
S2-C8	1.650(3)	50.86%	N5-Ni1-N3	87.92	47.56%
N5-N5	4.102(3)	1.95%	N5-Ni1-N6	92.23	43.25%
N5-C7	1.159(3)	1.01%	Ni1-N5-C7	158.03	22.56%
N6-C8	1.159	1.01%	Ni1-N6-C8	166.13	30.65%
S1-N5	2.804(4)	64.63%	N5-C7-S1	175.82	40.34%
S2-N6	2.809(3)	64.13%	N6-C8-S2	178.33	42.85%
			293 K		
Ni1-N5	2.054(2)	0.10%	N3-Ni1-N5	92.02	43.93%
Ni1-N6	2.037(2)	11.70%	N3-Ni1-N3	180.00	44.05%
S1-C7	1.642(2)	51.20%	N3-Ni1-N6	88.70	47.25%
S2-C8	1.637(3)	51.70%	N5-Ni1-N3	92.02	43.93%
N5-N5	4.108(3)	1.95%	N5-Ni1-N6	92.29	43.66%
N5-C7	1.151(3)	1.00%	Ni1-N5-C7	158.29	22.34%
N6-C8	1.164(3)	1.00%	Ni1-N6-C8	166.09	30.14%
S1-N5	2.792(3)	0.64%	N5-C7-S1	175.92	39.97%
S2-N6	2.801(3)	0.65%	N6-C8-S2	178.18	42.23%

Table S2. Hydrogen Bond Table of Compound **1** at Low and Room Temperatures

D-H···A	d(D-H)Å	d(H-A)Å	d(D-A)Å	$\lvert\delta = \bar{x} - \mu\rvert\%$	D-H···A(˚)	$\lvert\delta = \bar{x} - \mu\rvert\%$
			100 K			
N1-H1···S1	0.889	2.544	3.259	21.4%	137.85	5.08%
N1-H1···S2	0.889	2.674	3.288	18.5%	127.07	5.71%
C4-H5B···S2	0.990	2.798	3.735	26.3%	158.18	25.40%
C1-H1B···N4	0.990	2.793	3.484	1.15%	127.40	5.38%
C3-H3B···S1	0.990	2.915	3.723	25.10%	139.42	6.65%
C5-H5A···C5	0.990	2.924	3.346	12.65%	106.73	26.05%
			293 K			
N1-H1···S1	0.882	2.628	3.297	20.57%	133.36	0.29%
N1-H1···S2	0.882	2.651	3.304	19.87%	131.68	1.39%
C4-H5B···S2	0.990	2.800	3.743	24.03%	159.54	26.47%
C1-H1B···N4	0.990	2.838	3.523	2.03%	126.96	6.11%
C3-H3B···S1	0.990	2.947	3.754	25.13%	139.39	6.32%
C5-H5A···C5	0.990	2.963	3.395	10.77%	107.49	25.59%

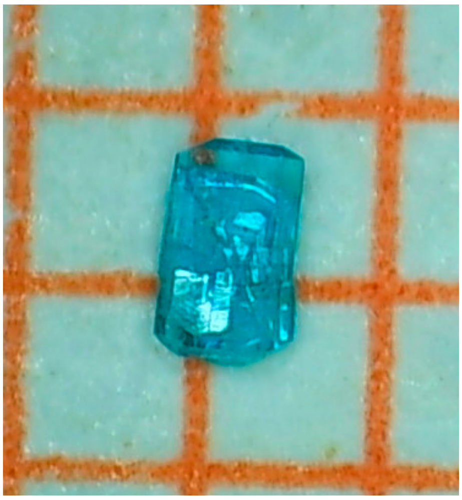

Figures S1. The appearance of compound **1**

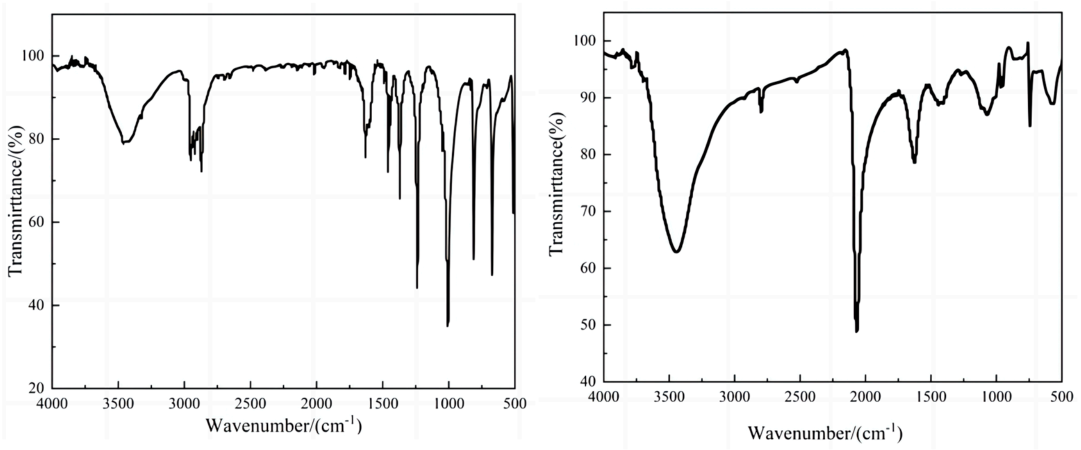

(a) (b)

Figures S2. (a) and (b) show the infrared spectra of raw materials Hmta and KSCN, respectively.

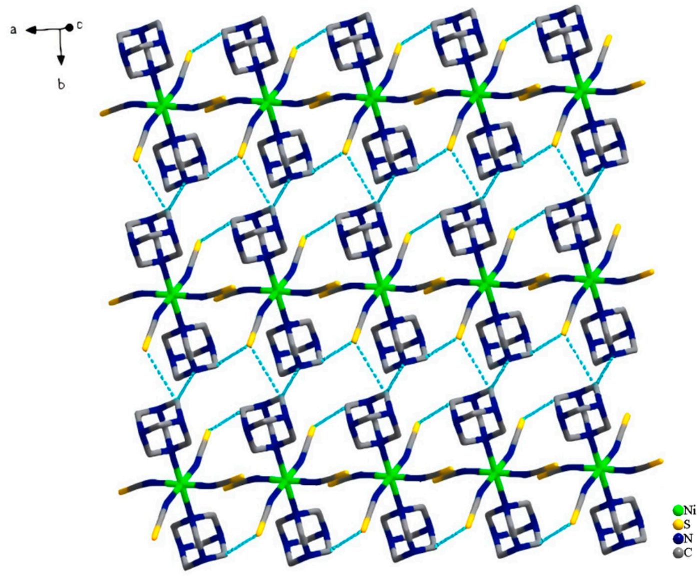

Figure S3. Arrangement diagram of compound **1**

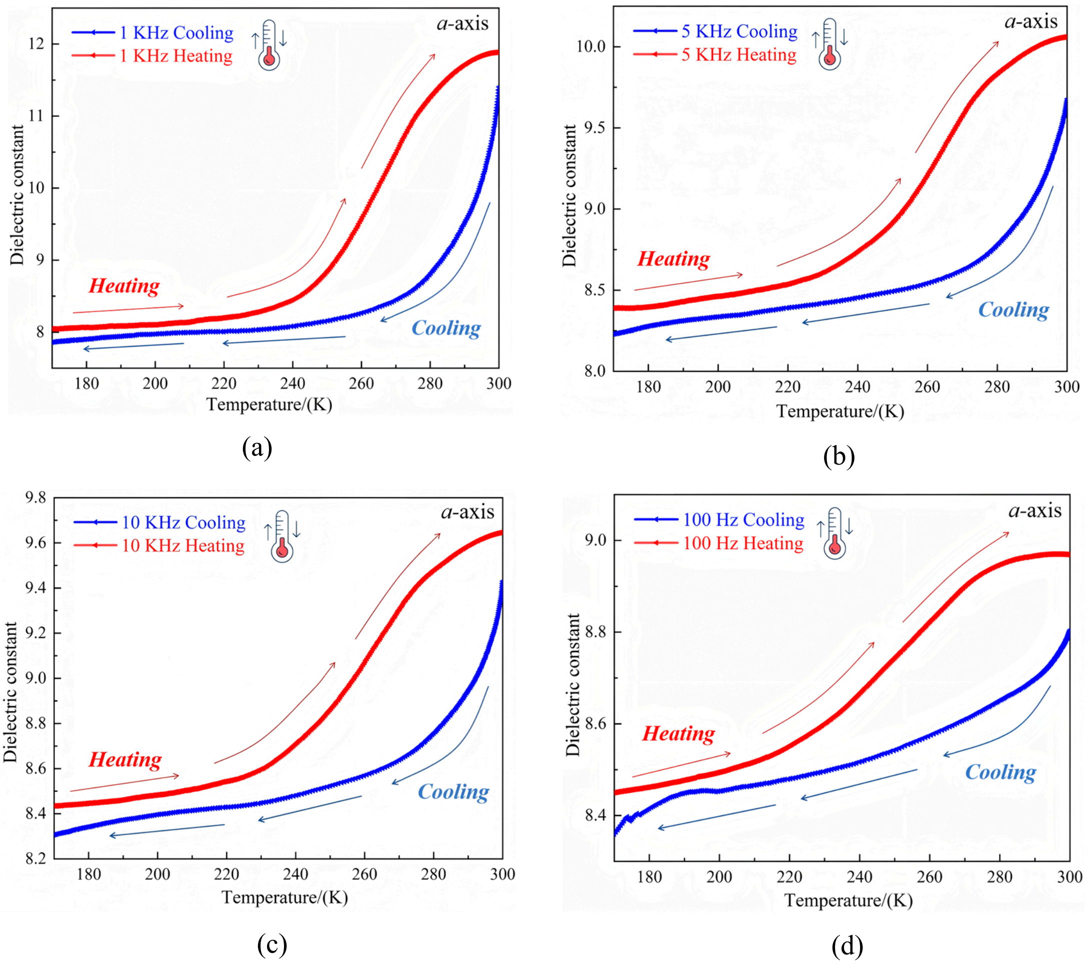

Figure S4. Shows the dielectric constant curves of compound **1** at various frequencies along the *a*-axis direction (a)、(b)、(c)、(d).

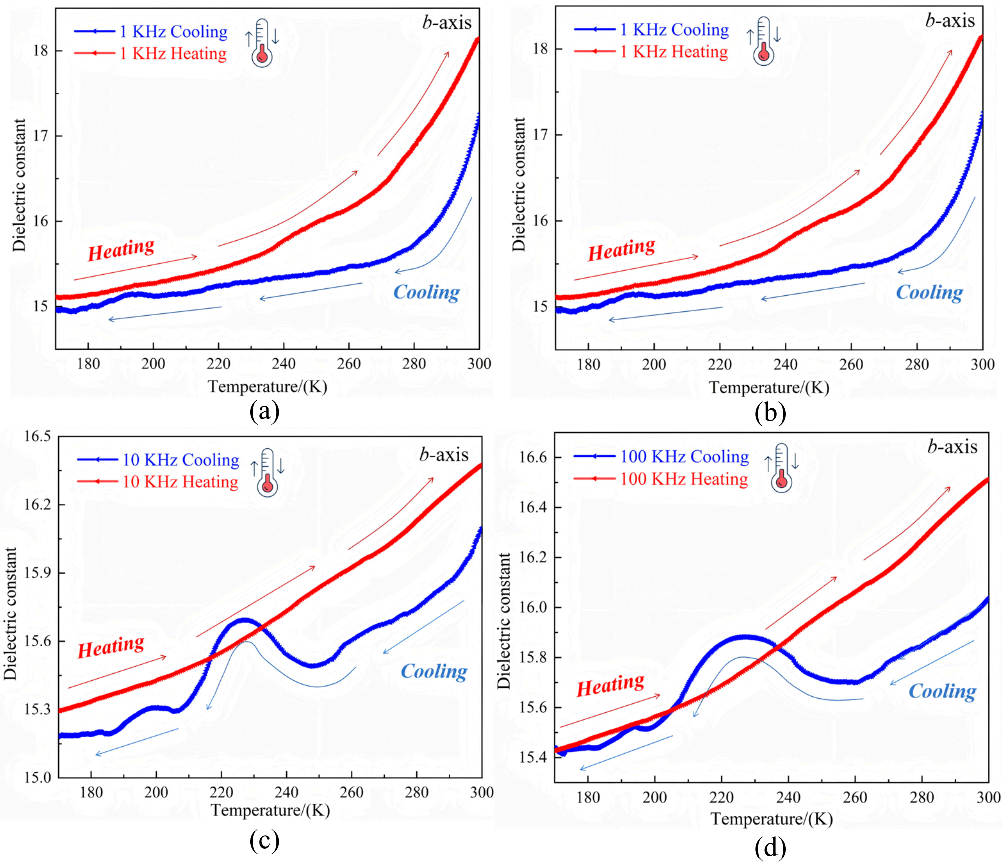

Figure S5. Shows the dielectric constant curves of compound **1** at various frequencies along the *b*-axis direction (a)、(b)、(c)、(d).

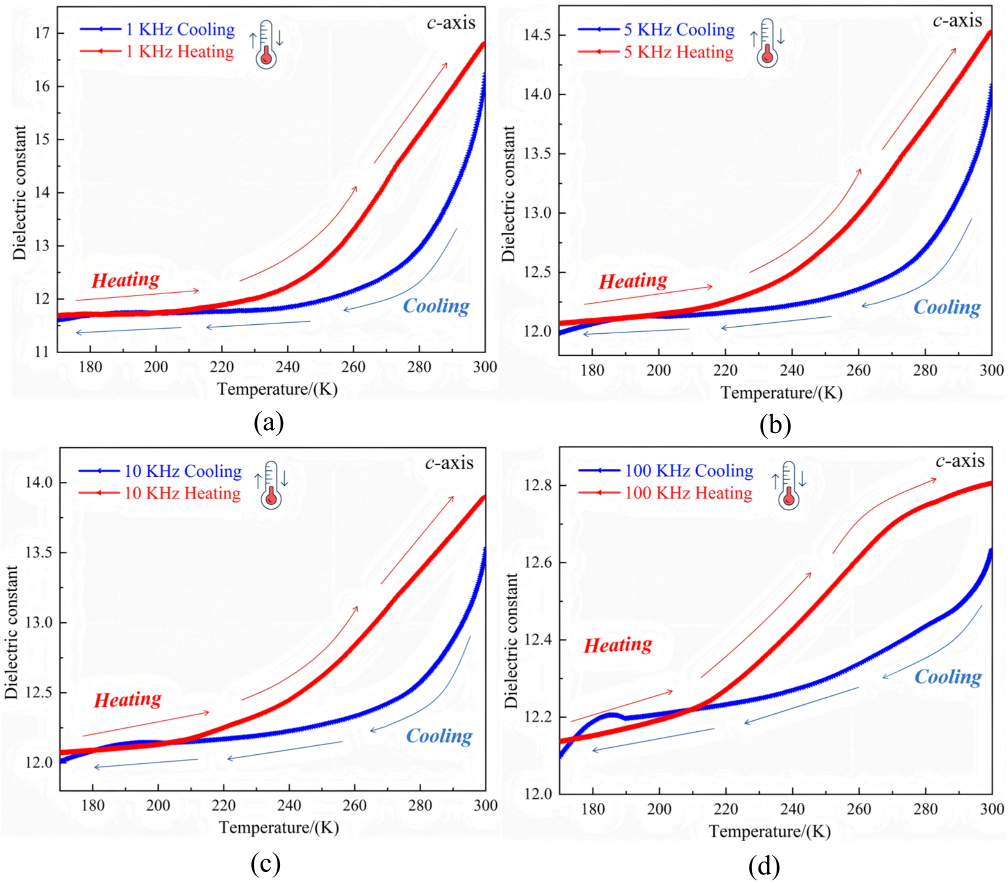

Figure S6. Shows the dielectric constant curves of compound **1** at various frequencies along the *c*-axis direction (a)、(b)、(c)、(d).

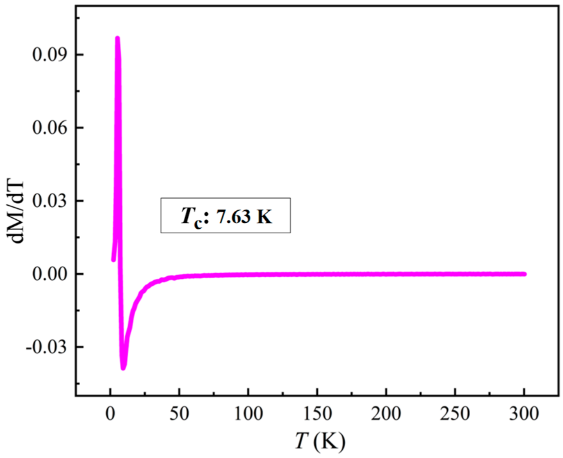

Figure S7. The Curie temperature of compound **1**
